# Structural basis for substrate recognition in the *Phytolacca americana* glycosyltransferase *Pa*GT3

**DOI:** 10.1107/S2059798322000869

**Published:** 2022-02-21

**Authors:** Rakesh Maharjan, Yohta Fukuda, Taisuke Nakayama, Toru Nakayama, Hiroki Hamada, Shin-ichi Ozaki, Tsuyoshi Inoue

**Affiliations:** a Graduate School of Pharmaceutical Science, Osaka University, Suita, Osaka 565-0871, Japan; b National Institute of Biomedical Innovation, Health and Nutrition, Ibaraki, Osaka 567-0085, Japan; c Department of Biomolecular Engineering, Graduate School of Engineering, Tohoku University, Sendai, Miyagi 980-8579, Japan; d Department of Life Science, Faculty of Science, Okayama University of Science, Okayama 700-0005, Japan; e Department of Biological Chemistry, Graduate School of Science and Technology for Innovation, Yamaguchi University, Yamaguchi 753-8515, Japan

**Keywords:** UGT, glycosylation, polyphenols, protein crystallization, capsaicin, kaempferol, crown ethers

## Abstract

Capsaicinoids are phenolic compounds that have health benefits. However, the pungency and poor water solubility of these compounds limit their exploitation. Glycosylation is a powerful method to improve water solubility and reduce pungency while preserving bioactivity. *Pa*GT3, a uridine diphosphate glycosyltransferase (UGT) from *Phytolacca americana*, is known for its ability to glycosylate capsaicinoids and other phenolic compounds. While structural information on several UGTs is available, structures of UGTs that can glycosylate a range of phenolic compounds are rare. To fill this gap, crystal structures of *Pa*GT3 with a sugar-donor analogue (UDP-2-fluoroglucose) and the acceptors capsaicin and kaempferol were determined. *Pa*GT3 adopts a GT-B-fold structure that is highly conserved among UGTs. However, the acceptor-binding pocket in *Pa*GT3 is hydrophobic and large, and is surrounded by longer loops. The larger acceptor-binding pocket in *Pa*GT3 allows the enzyme to bind a range of compounds, while the flexibility of the longer loops possibly plays a role in accommodating the acceptors in the binding pocket according to their shape and size. This structural information provides insights into the acceptor-binding mechanism in UGTs that bind multiple substrates.

## Abstract 2

Crystal structures of the UDP glycosyltransferase *Pa*GT3 in complex with various ligands are reported. This study sheds light on how the enzyme accommodates sugar acceptors of different shapes and sizes for successful glycosylation.

## 1. Introduction

Capsaicinoids are compounds with a pungent taste that are produced by plants belonging to the genus *Capsicum*. Despite contact with capsaicinoids causing the inflammation of tissue, these compounds also provide health benefits. Capsaicin shows a cardioprotective effect through the activation of transient receptor potential vanilloid 1 (TRPV1) and inhibition of platelet aggregation (Mittelstadt *et al.*, 2012 ▸; Sharma *et al.*, 2013 ▸). Capsaicin supplements in a high-fat diet lower adipose tissue weight and serum triglyceride in rats (Kawada *et al.*, 1986 ▸). Capsaicinoids also possess antibacterial (Marini *et al.*, 2015 ▸), anti-inflammatory (Kim *et al.*, 2003 ▸), anticancer (Clark & Lee, 2016 ▸), antioxidant (Rosa *et al.*, 2002 ▸) and analgesic effects (Fusco & Alessandri, 1992 ▸). However, the pungency and poor water solubility of capsaicinoids limit their use as prodrug compounds.

Uridine diphosphate glycosyltransferases (UGTs), which are classified as family 1 glycosyltransferases, transfer sugar moieties from UDP-sugar donors to small lipophilic molecules (Lombard *et al.*, 2014 ▸). Glycosylation of lipophilic molecules improves their water solubility, membrane permeability, cellular absorption and localization, and biological half-life (Bowles *et al.*, 2005 ▸). UGTs also play a significant role in the biosynthesis of secondary metabolites and the elimination of xenobiotic compounds (Brazier-Hicks *et al.*, 2007 ▸; Lim & Bowles, 2004 ▸; Radominska-Pandya *et al.*, 2010 ▸). Besides *in vivo* functions, UGTs have garnered attention for the one-step enzymatic glycosylation of small lipophilic compounds, compared with the chemical glycosylation method, which requires a tedious and long process of protection/deprotection of functional groups (Shimoda *et al.*, 2006 ▸; Dai *et al.*, 2017 ▸). Small-molecule glycosylation provides advantages in several biotechnological applications, such as increasing the water solubility of poorly water-soluble compounds such as resveratrol (Lepak *et al.*, 2015 ▸) and artepillin C (Shimoda *et al.*, 2014 ▸), improving the stability of vitamin C (Muto *et al.*, 1990 ▸), the production of indigo dye by an environmentally friendly process (Hsu *et al.*, 2018 ▸), synthesis of the skin whitener α-arbutin from hydroquinone (Kurosu *et al.*, 2002 ▸) and the producing of unnatural colours in flowers for decoration such as blue-coloured roses (Katsumoto *et al.*, 2007 ▸). Accordingly, glycosylation is one of the methods that are used to improve the water solubility and decrease the pungency of capsaicinoids (Kometani *et al.*, 1993 ▸). Capsaicinoid glycosides with improved solubility and reduced pungency show similar effects as the parent compounds and thus can find their way into preclinical trials as prodrugs.

UGTs share a conserved three-dimensional structure, known as a GT-B fold, consisting of two Rossmann-fold domains. These enzymes are characterized by the presence of a consensus plant secondary product glycosyltransferase (PSPG) motif, which contains most of the residues involved in UDP-sugar donor binding (Offen *et al.*, 2006 ▸; Lim & Bowles, 2004 ▸). The sugar-acceptor binding pocket, including the acceptor-recognizing residues, varies significantly among different UGTs, although a His–Asp catalytic pair is highly conserved. This variation in the acceptor-binding pocket could allow different UGTs to recognize different aglycones and glycosylate at different positions (Li *et al.*, 2007 ▸; Lairson *et al.*, 2008 ▸). To understand the structure–function relationship, several UGT crystal structures have been determined with or without substrates. Most of these UGT structures are in complexes with flavonoid molecules, such as *Vv*GT1 from *Vitis vinifera* with kaempferol/quercetin (Offen *et al.*, 2006 ▸), UGT78G1 from *Medicago truncatula* with myricetin (Modolo *et al.*, 2009 ▸) and UGT78K6 from *Clitoria ternatea* with delphinidin/petunidin/kaempferol (Hiromoto *et al.*, 2015 ▸). Recently, crystal structures of some UGTs that glycosylate other phenolic compounds have also been determined in complex with the corresponding acceptor substrates, such as UGT76G1 from *Stevia rebaudiana* with rebaudioside A/rubusoside (Yang *et al.*, 2019 ▸), *Pa*GT2 from *Phytolacca americana* with resveratrol/pterostilbene (Maharjan, Fukuda, Shimomura *et al.*, 2020 ▸) and *Os*79UGT from *Oryza sativa* with trichothecene (Wetterhorn *et al.*, 2017 ▸). Surprisingly, some UGTs can recognize and glycosylate a range of compounds. *Bs*-YjiC from *Bacillus subtilis* (Dai *et al.*, 2017 ▸) and UGT74AN1 from *Asclepias curassavica* (Wen *et al.*, 2018 ▸) glycosylate different classes of phenolic compounds. However, a lack of substrate-bound crystal structures of these promiscuous UGTs limits our understanding of acceptor recognition in such UGTs.

**Figure 1:**
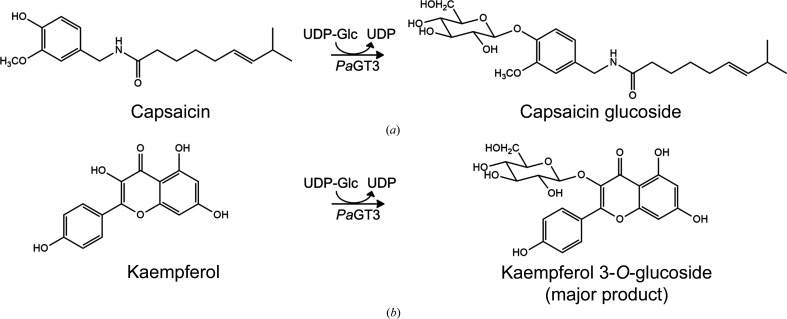
(*a*) *Pa*GT3 catalyzes the glycosylation of capsaicin to form capsaicin glucoside. (*b*) With kaempferol, *Pa*GT3 form a mixture of kaempferol glucosides, with kaempferol 3-*O*-glucoside as the major product.

*P. americana* is a toxic plant that is native to North America. Previously, three UGTs from the plant, namely *Pa*GT1–*Pa*GT3, have been isolated and characterized (Noguchi *et al.*, 2009 ▸). These UGTs could be involved in the biosynthesis of flavonoid and/or triterpene glycoside derivatives, which have been isolated from different parts of *P. americana* (Bylka & Materławska, 2001 ▸; Takahashi *et al.*, 2001 ▸; Wang *et al.*, 2008 ▸). Among these three UGTs, *Pa*GT3 is capable of glycosylating capsaicinoids (Fig. 1 ▸; Noguchi *et al.*, 2009 ▸). Moreover, it has been shown that capsaicin can increase the expression of *Pa*GT3 in cultured callus tissue of *P. americana*. Additional studies show that *Pa*GT3 glycosylates a range of substrates which includes flavonols, stilbenoids, hydroxyflavones, hydroxybenzoic acids, retinol, vitamin E and its derivatives, and artepillin C (Iwakiri, Imai *et al.*, 2013 ▸; Ozaki *et al.*, 2012 ▸; Shimoda *et al.*, 2006 ▸, 2014 ▸; Iwakiri, Mase *et al.*, 2013 ▸). However, *Pa*GT3 forms multiple monoglycosylated products, rendering low regioselectivity with compounds that have more than one glycosylation site, such as kaempferol, artepillin C and resveratrol (Noguchi *et al.*, 2009 ▸; Shimoda *et al.*, 2014 ▸; Ozaki *et al.*, 2012 ▸).

Although crystal structures of the promiscuous UGTs *Pa*GT3 (Maharjan, Fukuda, Nakayama *et al.*, 2020 ▸) and *Bs*-YjiC (Dai *et al.*, 2021 ▸) are available, these structures do not contain acceptors. Thus, these structures do not provide sufficient information to understand the acceptor-recognition mechanisms in such promiscuous UGTs. Similarly, capsaicin glycosides have been enzymatically synthesized using different cultured plant cells (Shimoda *et al.*, 2007 ▸; Katsuragi *et al.*, 2010 ▸, 2011 ▸). However, the enzyme or UGT that transforms capsaicin in these plant cell cultures is not known and structural information is not available. Thus, to shed light on the mechanism of capsaicin glycosylation and the acceptor-recognition mechanism in a promiscuous UGT, we report crystal structures of *Pa*GT3 in complex with the sugar-donor analogue uridine-2-fluoroglucose (UDP-2FGlc) at 2.20 Å resolution as well as of *Pa*GT3 with UDP-2FGlc and capsaicin at 2.60 Å resolution. We also determined the crystal structure of *Pa*GT3 with UDP-2FGlc and kaempferol at 1.85 Å resolution to understand the poor regioselectivity in the glycosylation of acceptors with multiple possible glycosylation sites. The structure of *Pa*GT3 with capsaicin provides a mechanistic overview of the recognition of long-chain phenolic compounds in UGTs, while the structure of the kaempferol complex elaborates the poor regioselective glycosylation of phenolic compounds with multiple possible glycosylation sites.

## 2. Materials and methods

### 2.1. Protein expression and purification

The gene expressing *Pa*GT3 was cloned and expressed and the protein was purified as described previously (Maharjan, Fukuda, Nakayama *et al.*, 2020 ▸). Briefly, the gene encoding *Pa*GT3 (UniProt ID B5MGN9) was amplified by polymerase chain reaction (PCR) using the forward and reverse primers 5′-CTTTATTTCCAGGGTATGGGTGCTGAACCTCAACAG-3′ and 5′-AGCAGAGATTACCTAAGCATGATAACCCCTCAACTCCTC-3′, respectively. The obtained product was ligated into a modified pCold I vector which contained a Tobacco etch virus (TEV) protease site after the hexahistidine sequence (His_6_ tag). The protein was overexpressed in *Escherichia coli* strain BL21 (DE3) by induction with 0.4 m*M* isopropyl β-d-1-galactopyranoside (IPTG) for 24 h at 15°C. The cells were collected by centrifugation and resuspended in buffer consisting of 20 m*M* Tris–HCl pH 8.5, 100 m*M* NaCl, 5 m*M* dithiothreitol (DTT), 10 m*M* imidazole including protease-inhibitor cocktail (Roche). The cells were lysed by sonication and were then centrifuged to remove cell debris. The supernatant was loaded onto a nickel–nitrilotriacetic acid (Ni–NTA) column (HisTrap HP 5 ml, GE Healthcare) and the target protein was eluted using a buffer consisting of 20 m*M* Tris–HCl pH 8.5, 100 m*M* NaCl, 5 m*M* DTT, 300 m*M* imidazole. The fraction containing *Pa*GT3 was pooled, mixed with TEV protease and dialyzed against 20 m*M* Tris–HCl pH 8.5, 100 m*M* NaCl, 5 m*M* DTT. The dialysed protein was passed through an Ni–NTA column to separate *Pa*GT3 from the His_6_ tag and TEV protease. The protein was further purified by cation-exchange chromatography on a HiTrap Q column (GE Healthcare) and eluted with a buffer consisting of 20 m*M* Tris–HCl pH 8.5, 1 *M* NaCl. Finally, size-exclusion chromatography (SEC) was performed on a HiLoad 16/600 Superdex 200 pg column using a buffer consisting of 20 m*M* Tris–HCl pH 8.0, 100 m*M* NaCl, 5 m*M* dithiothreitol. The fractions containing *Pa*GT3 were pooled, concentrated to ∼10 mg ml^−1^ and stored at −80°C until crystallization. Macromolecule-production information is summarized in Table 1 ▸.

### 2.2. Crystallization

For crystallization experiments, UDP-2FGlc was purchased from Fuji Molecular Planning, Yokohama, Japan. Crystallization screening of *Pa*GT3 with UDP-2FGlc and/or acceptors was performed in a 96-well sitting-drop crystallization plate (Violamo) using an automatic pipetting machine (Mosquito LCP, TTP Labtech). As for apo *Pa*GT3, the co-crystallization of *Pa*GT3 with ligands did not form diffracting crystals without 18-crown-6 ether. For crystallization screening, 100 nl protein solution consisting of 10 mg ml^−1^ *Pa*GT3, 50 m*M* 18-crown-6 ether, 5 m*M* UDP-2FGlc, 2 m*M* capsaicin or kaempferol was mixed with 100 nl reservoir solution. Crystals appeared overnight using a reservoir solution consisting of 0.2 *M* potassium acetate, 20%(*w*/*v*) PEG 3350.

For diffraction experiments, crystals of *Pa*GT3 with UDP-2FGlc were obtained by mixing 1 µl protein solution consisting of 10 mg ml^−1^ *Pa*GT3, 50 m*M* 18-crown-6 ether, 5 m*M* UDP-2FGlc with 1 µl reservoir solution consisting of 0.15–0.20 *M* potassium acetate, 20%(*w*/*v*) PEG 3350. Cystals of *Pa*GT3 with UDP-2FGlc and kaempferol were obtained by mixing 1 µl protein solution consisting of 10 mg ml^−1^ *Pa*GT3, 50 m*M* 18-crown-6 ether, 5 m*M* UDP-2FGlc, 2 m*M* kaempferol with 1 µl reservoir solution consisting of 0.15–0.20 *M* potassium acetate, 20%(*w*/*v*) PEG 3350. Although crystals of *Pa*GT3 with UDP-2FGlc and capsaicin (2–10 m*M*) were obtained under similar conditions, electron density for capsaicin was not observed during structure determination. Thus, to determine the capsaicin-bound *Pa*GT3 structure, crystals of *Pa*GT3 with UDP-2FGlc were soaked in reservoir solution containing an excess of capsaicin before harvesting. For data collection, all crystals were harvested by soaking in a cryoprotectant solution consisting of the reservoir solution supplemented with 15% ethylene glycol. Crystallization information is summarized in Table 2 ▸.

### 2.3. Data collection and structure determination

All data sets were collected on beamline BL44XU at SPring-8, Japan. The data sets were processed with the *XDS* package (Kabsch, 2010 ▸) and were scaled with *AIMLESS* (Evans, 2011 ▸) in the *CCP*4 package (Winn *et al.*, 2011 ▸). The phases for each structure were determined by molecular replacement in *MOLREP* (Vagin & Teplyakov, 2010 ▸) using the structure of apo *Pa*GT3 (PDB entry 6lzy; Maharjan, Fukuda, Nakayama *et al.*, 2020 ▸) as the search model. *Coot* (Emsley *et al.*, 2010 ▸) was used for manual model building, adding substrates into the corresponding electron-density maps and adding water molecules. The polder omit map for capsaicin was calculated using *phenix.polder* (Liebschner *et al.*, 2017 ▸) in *Phenix* (Liebschner *et al.*, 2019 ▸). Refinement was performed in *REFMAC*5 (Kovalevskiy *et al.*, 2018 ▸) and *phenix.refine*. The structures were validated using *MolProbity* (Williams *et al.*, 2018 ▸). Images were prepared using *PyMOL* (version 1.8; Schrödinger). The data-collection and refinement statistics are given in Tables 3 ▸ and 4 ▸.

## 3. Results

### 3.1. The overall structure of *Pa*GT3 complexes

**Figure 2:**
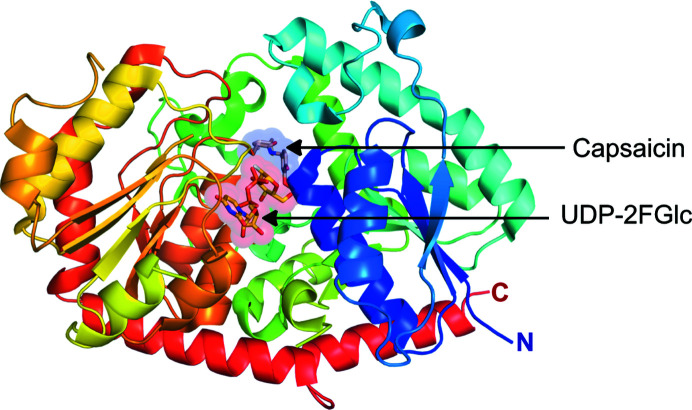
Overall structure of substrate-bound *Pa*GT3. Crystal structure of *Pa*GT3 colour-ramped from the N-terminus (blue) to the C-terminus (red). The donor (UDP-2FGlc, yellow sticks) and acceptor (capsaicin, wheat sticks) binding sites are highlighted in transparent red and blue colours, respectively.

Recombinant *Pa*GT3 was expressed and purified to near-homogeneity for crystallization as described previously (Maharjan, Fukuda, Nakayama *et al.*, 2020 ▸). The crystal structure of *Pa*GT3 with the sugar-donor analogue UDP-2FGlc was determined at 2.20 Å resolution (Supplementary Fig. S1*a* ). The ternary complexes of *Pa*GT3 with UDP-2FGlc and the sugar acceptors capsaicin or kaempferol were refined to 2.60 and 1.85 Å resolution, respectively (Fig. 2 ▸ and Supplementary Figs. S1*b* and S1*c* ). The asymmetric unit of each crystal structure of *Pa*GT3 consists of two molecules of the enzyme linked together with an 18-crown-6 metal-ion complex, which plays the role of a molecular glue during crystallization (Maharjan, Fukuda, Nakayama *et al.*, 2020 ▸). Data-collection and refinement statistics are given in Tables 3 ▸ and 4 ▸, respectively.

The ligand-bound *Pa*GT3 structures are nearly identical to the apo *Pa*GT3 structure (Supplementary Fig. S2). Structural alignment of apo *Pa*GT3 with the complexes of *Pa*GT3 with UDP-2FGlc, with UDP-2FGlc and capsaicin, and with UDP-2FGlc and kaempferol shows root-mean-sqaure deviations (r.m.s.d.s) of 0.71, 0.70 and 0.89 Å, respectively, for all C^α^ atoms. However, closer examination shows the displacement of some loops that are present around the substrate-binding cavity. Compared with the apo-*Pa*GT3 structure, the loop Gly78–Gly91 shifts towards the acceptor-binding pocket in the kaempferol-bound structure (Supplementary Fig. S2). Compared with the apo *Pa*GT3 structure the same loop is seen to shift outwards in the UDP-2FGlc-bound structure, and it shifts further outwards in the capsaicin-bound structure due to the binding of the larger capsaicin molecule. The loops Cys289–Ile297 and Val412–Lys429 shift towards the pocket in the substrate-bound structures compared with the apo *Pa*GT3 structure. These results show that *Pa*GT3 adopts similar conformations with subtle differences to accommodate acceptors with different shapes and sizes.

The crystallization of *Pa*GT3 requires 18-crown-6 ether as a crystallization additive. Similar to the crystal structure of apo *Pa*GT3, a crown ether molecule is present between the two protomers of the protein in the asymmetric unit (Supplementary Fig. S1). The crown ether cavity consists of a metal ion coordinated through the six O atoms of the crown ether and the main-chain O atoms of Glu238 from the two molecules of *Pa*GT3. Previously, we assigned the metal ion in the crown ether cavity as a sodium ion, because the apo *Pa*GT3 crystallization solution contained sodium bromide. *Pa*GT3–substrate complex crystals were obtained with a mother-liquor solution containing potassium acetate. Usually, the distances between potassium and oxygen in macromolecular crystal structures are >2.7 Å, while sodium–oxygen distances are between 2.4 and 2.5 Å (Zheng *et al.*, 2017 ▸). In the *Pa*GT3–capsaicin crystal structure the average distances from the central metal ion to the O atoms and C atoms of the 18-crown-6 ether are 2.9 and 3.6 Å, respectively. These values are comparable to the K–O and K–C distances reported in the crystal structure of a 18-crown-6 ether–potassium ion complex (Ozutsumi *et al.*, 1989 ▸). The distances from the central potassium ion to the O and C atoms of the crown ether in the *Pa*GT3–capsaicin crystal structure are presented in Supplementary Table S2. Moreover, it is known that 18-crown-6 ether has a higher affinity for potassium ion than for sodium ion. Thus, we assign the electron density in the crown ether cavity present in the *Pa*GT3 complex structures as a potassium ion, which could be from the crystallization solution.

### 3.2. Sugar-donor binding in *Pa*GT3

**Figure 3:**
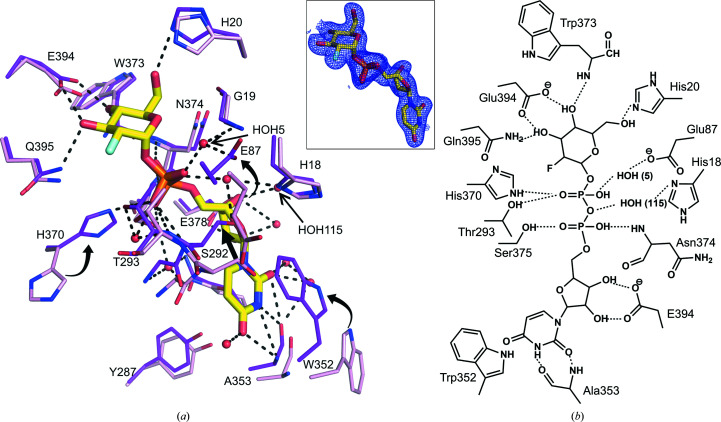
Interaction between *Pa*GT3 and UDP-2FGlc. (*a*) Residues of *Pa*GT3 (magenta) from the kaempferol/UDP-2FGlc-bound structure showing the sugar-donor analog stabilized by a network of hydrogen bonds. Side chains of the corresponding residues in apo *Pa*GT3 (pink) show that the residues around UDP-2FGlc shift towards the substrate, where the side chains of Glu87, Ser292, Trp352 and His370 shows large movements. Water molecules involved in the hydrogen-bond network are shown as red spheres. The possible hydrogen bonds are indicated with dashed lines. The inset shows a σ-weighted 2*F*_o_ − *F*_c_ electron-density map contoured at 1σ for UDP-2FGlc in the *Pa*GT3/UDP-2FGlc/kaempferol structure. (*b*) 2D figure showing residues that interact with UDP-2FGlc in the crystal structure.

The C-terminal domain harbours the sugar-donor binding cavity in GT-B-fold UGTs. UDP-2FGlc occupies the sugar-donor binding cavities in all three *Pa*GT3 crystal structures (Supplementary Fig. S1). Electron density for UDP-2FGlc is present in all three *Pa*GT3 structures (Fig. 3 ▸ *a*). Among our three substrate-bound *Pa*GT3 structures, the UDP-2FGlc/kaempferol-containing structure has the highest resolution. Thus, we describe the features of UDP-2FGlc binding in *Pa*GT3 with reference to this structure. The residues that interact with UDP-2FGlc mainly come from the C-terminal domain and are shown in Fig. 3 ▸(*b*). Most of these sugar-donor-recognizing residues are highly conserved in the GT-B-fold UGTs and come from the consensus PSPG motif, which extends from Trp352 to Gln395 in *Pa*GT3 (Supplementary Fig. S3). Hence, the sugar-donor binding in *Pa*GT3 is comparable to other that in known plant UGT structures and has been discussed in a previous report.

Among the residues interacting with UDP-2FGlc, the side chains of Ser292, Trp352 and His370 show different configurations when compared with the apo *Pa*GT3 structure (Fig. 3 ▸ *c*). Although Ser292 is outside the PSPG motif, it is seen to form hydrogen bonds with the sugar-donor analogue. The movement of Ser292 comes from movement of the Cys289–Ile297 loop in substrate-bound structures (Supplementary Fig. S2). In the substrate-bound *Pa*GT3 structures, the indole moiety of Trp352 flips ∼180° to form a π-stacking interaction with the uracil ring of the sugar-donor analogue. Such a π-stacking interaction between the uridine moiety and the corresponding Trp residue has been observed in several UGT structures (Hiromoto *et al.*, 2015 ▸; Brazier-Hicks *et al.*, 2007 ▸; Yang *et al.*, 2019 ▸). Similarly, the side chain of His370 rotates ∼120° to form a hydrogen bond to the O3A atom on the α-phosphate moiety of UDP-2FGlc. This histidine residue is highly conserved among UGTs and plays a remarkable role in sugar-donor binding. For example, mutation of His293 in the *Streptomyces antibioticus* UGT OleI (Bolam *et al.*, 2007 ▸), corresponding to His370 in *Pa*GT3, significantly diminishes the activity of the enzyme.

In addition to the residues from the C-terminal domain, UDP-2FGlc in *Pa*GT3 structures is also stabilized through hydrogen bonds from residues in the N-terminal domain (Fig. 3 ▸ *c*). The side chain of His18 is likely to contribute to stabilizing the sugar moiety by forming a hydrogen bond to the 6-OH of 2-fluoroglucose. His18, Gly19 and Glu87 stabilize the sugar-donor analogue through water-mediated hydrogen bonds. Interestingly, the side chain of Glu87 makes a large movement to form a hydrogen bond to a water molecule (HOH5) in the substrate-bound structure. The movement of Glu87 is a result of the shift of the Gly78–Gly91 loop in the substrate-bound structures (Supplementary Fig. S2).

### 3.3. Sugar-acceptor binding in *Pa*GT3

The ternary complexes of *Pa*GT3 with UDP-2FGlc and aglycones were prepared by either soaking or co-crystallization methods. For the capsaicin-bound complex, co-crystals of *Pa*GT3 and UDP-2FGlc were soaked in reservoir solution containing an excess of capsaicin, whereas *Pa*GT3 was co-crystallized with UDP-2FGlc and kaempferol to obtain the ternary complex with kaempferol.

Among the two protomers of *Pa*GT3 in the asymmetric unit, molecule *A* does not show any possible electron density for capsaicin in the acceptor-binding site. However, molecule *B* shows an elongated *mF*_o_ − *DF*_c_ electron density in the acceptor-binding pocket. Initially, the electron-density map was not clear enough to determine a capsaicin molecule (Supplementary Fig. S4*a* ). However, this electron density is large than an ethylene glycol molecule, which was used as a cryoprotectant. We assumed that the electron density is from a bound capsaicin molecule and that the poor electron density could possibly be due to low occupancy or/and the highly flexible alkyl chain of capsaicin. Thus, we modelled a capsaicin molecule in the acceptor-binding site in *Pa*GT3 molecule *B* and refined it to an occupancy of 0.8. To confirm the presence of capsaicin, we calculated a *mF*_o_ − *DF*_c_ polder omit map (Liebschner *et al.*, 2017 ▸) in the *Phenix* suite, which excludes bulk solvent from the selected area to calculate the omit map (Fig. 3 ▸ *a*). The calculated polder map confirms the occupancy of capsaicin in *Pa*GT3 molecule *B*. The 2*mF*_o_ − *DF*_c_ electron-density map contoured at 1σ for capsaicin is comparable to the calculated polder map (Supplementary Fig. S4*b* ). We also added a capsaicin molecule in the acceptor-binding pocket of molecule *A* and similarly calculated an *mF*_o_ − *DF*_c_ polder map for it. However, the program is unable to calculate an interpretable omit map for capsaicin in *Pa*GT3 molecule *A*, suggesting the absence of capsaicin in protomer *A*.

**Figure 4:**
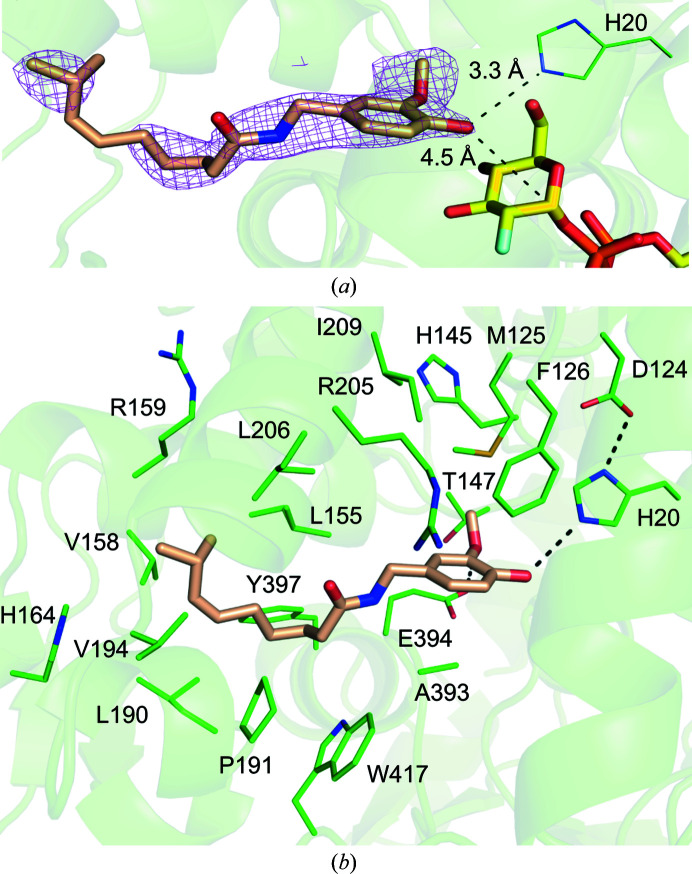
Capsaicin binding in *Pa*GT3. (*a*) *mF*_o_ − *DF*_c_ polder map (purple mesh) for capsaicin (wheat sticks) contoured at 5σ. The distances from the putative glycosylation site on capsaicin to the catalytic histidine (His20) and the C1 carbon of UDP-2FGlc (yellow sticks) are indicated. (*b*) The interaction between capsaicin and *Pa*GT3 residues shows that the acceptor is mainly stabilized through the hydrophobic interactions in the acceptor-binding pocket.

According to the calculated polder map, the 10-OH group of capsaicin, which is the putative glycosylation site, faces towards the catalytic histidine His20 (Fig. 4 ▸ *b*). The distance from His20 to the 10-OH of capsaicin is 3.3 Å. The GT-B-fold UGTs contain a conserved His–Asp catalytic pair. From the *Pa*GT3 crystal structures as well as from comparison with other UGTs, the His20–Asp124 pair has been identified as the conserved catalytic pair in *Pa*GT3. Moreover, the mutation of His20 to Ala or Asp has been shown to completely impair the activity of the enzyme (Ozaki *et al.*, 2012 ▸). Another UGT from *P. americana*, *Pa*GT2, has been shown to possess two catalytic histidines: the conserved catalytic histidine His18 and the alternate catalytic residue His81 (Maharjan, Fukuda, Shimomura *et al.*, 2020 ▸). The mutation of either of the catalytic histidines in *Pa*GT2 was compensated by another catalytic histidine, which helped to retain the catalytic activity of the enzyme. However, no such residue that can catalyse glycosylation in the absence of His20 is observed around capsaicin in *Pa*GT3.

Within 4.5 Å, capsaicin is mainly surrounded by hydrophobic side-chain and a few polar side-chain amino acids. These residues include His20, Met125, Phe126, His145, Thr147, Leu155, Val158, His164, Leu190, Pro191, Val194, Leu206, Ala393, Glu394, Tyr397 and Trp417. The shortest distances between the atoms of these residues and the atoms of capsaicin are listed in Supplementary Table S2. Although Arg205 and Ile209 are farther from capsaicin, the side chains of these residues are also involved in formation of the acceptor-binding site. The phenolic ring of capsaicin is stacked between the side chains of Met125-Phe126 and Ala393-Glu394. A hydrophobic cavity formed by the side chains of Leu155, Val158, Arg159, His164, Leu190, Pro191, Val194, Leu206, Tyr397 and Trp417 harbours the alkyl chain of capsaicin. Although the acceptor-binding pocket is formed by numerous residues, capsaicin can only possibly form hydrogen bonds to His20 and Glu394. This suggests that capsaicin and other acceptor molecules in the acceptor-binding pocket of *Pa*GT3 are mainly stabilized by hydrophobic interactions. As capsaicin contains a single glycosylation site, it forms only a single glycosylated product. However, compounds such as artepillin C contain two possible glycosylation sites and *Pa*GT3 can form both artepillin C 4-β-d-glucoside and artepillin C 9-β-d-glucoside (Shimoda *et al.*, 2014 ▸). This suggests that due to the lack of extensive hydrogen bonds between the enzyme and acceptor molecules, *Pa*GT3 can recognize a single acceptor molecule in different binding orientations to form multiple possible products. This observation is comparable to the sugar-acceptor binding pocket in *S. rebaudiana* UGT76G1, which also recognizes acceptor molecules through hydrophobic interactions and recognizes a single steviol acceptor molecule in different orientations to form different products (Yang *et al.*, 2019 ▸).

**Figure 5:**
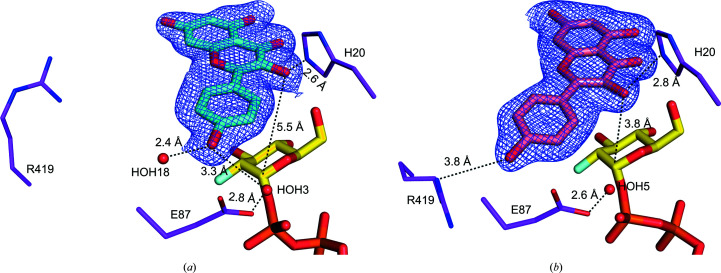
Kaempferol binding in *Pa*GT3. σ-Weighted 2*mF*_o_ − *DF*_c_ electron-density maps (blue mesh) contoured at 1σ for kaempferol in (*a*) molecule *A* (cyan sticks) and (*b*) molecule *B* (salmon sticks) in the asymmetric unit of the crystal structure of *Pa*GT3. The distances from kaempferol to nearby residues of *Pa*GT3, the C1 carbon of UDP-2FGlc and water molecules (red spheres) are indicated.

The crystal structure of *Pa*GT3 with UDP-2FGlc and kaempferol is similar to the capsaicin-complexed structure, with an r.m.s.d. of 0.34 Å for 855 C^α^ atoms. The electron density of kaempferol in both molecules of the enzyme clearly indicates that ring A faces towards the inner side of the acceptor-binding pocket (Figs. 5 ▸ *a* and 5 ▸ *b*). Superimposition of the two molecules of *Pa*GT3 in the asymmetric unit shows that the structures of these two protomers are highly similar (r.m.s.d. of 0.3 Å for 375 C^α^ atoms). The acceptor-binding pocket in *Pa*GT3 appears to be large for a kaempferol molecule. Thus, in addition to kaempferol, some ethylene glycol molecules from the cryoprotectant solution and water molecules are present in the acceptor-binding pocket (Supplementary Fig. S5). Superposition of the kaempferol molecules in molecules *A* and *B* of the enzyme shows that the binding positions of the two kaempferol molecules are different (Supplementary Fig. S6*b* ). In molecule *A* the 5-OH of kaempferol forms a hydrogen bond to Lys210 via a water molecule (HOH114). Due to the shift of kaempferol in molecule *B* and the absence of a water molecule, no such hydrogen bond is observed between the 5-OH of kaempferol and Lys210 (Supplementary Fig. S5). The 3-OH of kaempferol in molecule *A* can form a hydrogen bond to the main-chain carbonyl O atom of His145. In molecule *B*, the 3-OH of kaempferol is placed farther away from His145. Similarly, the 4′-OH of kaempferol in molecule *A* is stabilized by hydrogen bonds to two water molecules (HOH3 and HOH18). The side chain of Arg419 in molecule *A* flips away from kaempferol. In molecule *B*, Arg419 flips towards the kaempferol molecule and occupies a position relative to the water molecule (HOH18) to form a hydrogen bond to the 4′-OH of kaempferol. The importance of Arg419 is not known; however, this residue seems to be important for binding smaller aglycones such as kaempferol for stabilization and glycosylation by *Pa*GT3. While a water molecule (HOH5) occupies the corresponding position to HOH3, this water molecule is too distant from the 4′-OH to form a hydrogen bond. Interestingly, the 3-OH groups on kaempferol are at distances of 2.6 and 2.9 Å from the catalytic His20 in molecules *A* and *B* of *Pa*GT3, respectively. However, the distances between 3-OH of kaempferol and the C1 atom in the 2FGlc moieties of UDP-2FGlc in molecules *A* and *B* of *Pa*GT3 are 5.5 and 3.8 Å, respectively. As *Pa*GT3 glycosylates kaempferol to form kaempferol 3-*O*-glucoside as the major product, the binding orientation of kaempferol in molecule *B* is likely to be a close representation of the Michaelis complex that forms the major product. The binding of kaempferol in *Pa*GT3 protomers in the asymmetric unit is an indication that sugar-acceptor molecules can bind in different orientations in the acceptor-binding pocket. Hence, *Pa*GT3 forms more than one glycosylated product with sugar acceptors containing more than one glycosylation site (Noguchi *et al.*, 2009 ▸; Shimoda *et al.*, 2014 ▸).

Although the structures of *Pa*GT3 with capsaicin and kaempferol show high similarity, there is a slight difference in their acceptor-binding pockets. Mainly, the loops Gly78–Gly91 and Val412–Lys429 are seen to shift towards the acceptor-binding site in the kaempferol-bound structure (Supplementary Fig. S2). This could be due to differences in the binding orientations as well as in the shapes and sizes of capsaicin and kaempferol. In addition to the residues binding capsaicin, the other residues Ala17, Gly19, Glu87, Leu86, Phe99, Phe392 and Arg419 also take part in forming the acceptor-binding pocket for kaempferol. The involvement of these extra residues in the stabilization of kaempferol is evident from the movement of the loops (mentioned above) towards the active site compared with the capsaicin-bound structure (Supplementary Fig. S2).

In the kaempferol-bound structure, some extra electron density is present in the acceptor-binding pocket. We modelled this electron density as molecules of ethylene glycol, which was used as a cryoprotectant (Supplementary Fig. S6*a* ). The structural alignment of capsaicin- and kaempferol-bound *Pa*GT3 structures shows that one of the ethylene glycol molecules aligns with the alkyl chain of capsaicin (Supplementary Fig. S6*c* ). Similarly, the space occupied by the terminal methyl groups of capsaicin is occupied by two water molecules in the kaempferol-bound structure. The putative10-OH glycosylation site of capsaicin also aligns with the 3-OH of kaempferol in *Pa*GT3 molecule *B*, although the 10-OH of capsaicin is a little away from the catalytic residue His20. This indicates that the orientation of capsaicin calculated by the polder maps is in good agreement. Thus, the capsaicin-bound *Pa*GT3 structure shows that the enzyme can bind and catalyse the glycosylation of large phenolic compounds. However, due to the large acceptor-binding pocket and the lack of residues that can stabilize acceptor molecules with hydrogen bonds, *Pa*GT3 shows relatively low glycosylation activity towards smaller molecules such as salicyl acid, *trans*-*p*-coumaric acid and *m*-hydroxybenzoic acid compared with the larger capsaicin or kaempferol molecules (Noguchi *et al.*, 2009 ▸).

### 3.4. Catalytic mechanism of *Pa*GT3

Similar to other plant UGTs, *Pa*GT3 is an inverting glycosyltransferase that belongs to the GT1 family in the Carbohydrate Active Enzymes database. In these UGTs, glycosylation is catalysed by a conserved His–Asp pair in the active site. The highly conserved histidine residue acts as a catalytic base to remove the proton from the glycosylation site on the acceptor and the aspartate is thought to stabilize the protonated catalytic histidine. The generated nucleophilic acceptor then attacks the C1 carbon of the UDP-sugar to form the product, with displacement of UDP.

In the crystal structures of *Pa*GT3 the putative glycosylation sites of capsaicin and kaempferol are close to His20–Asp124 pair. Structural and amino-acid sequence alignment show His20–Asp119 in *Vv*GT1, His18–Asp115 in *Pa*GT2, His25–Asp124 in UGT76G1 and His17–Asp114 in UGT78K6 to occupy equivalent positions. In the *Pa*GT3–capsaicin structure the 10-OH group of capsaicin is about 3.3 and 4.5 Å away from the N atom of His20 and the C1 carbon of UDP-2FGlc, respectively. Similarly, the 3-OH group of kaempferol in molecule *B* of the *Pa*GT3–kaempferol crystal structure is about 2.8 and 3.8 Å away from His20 and the C1 carbon of UDP-2FGlc, respectively. In a previous study, His20Ala mutant *Pa*GT3 failed to form glycosylated products. These results suggest His20 to be the catalytic base that abstracts a proton from the acceptor molecule to generate a nucleophile, which then attacks the C1 atom of UDP-glucose to form products. Asp119 is the possible catalytic pair which stabilizes the protonated His20.

### 3.5. Comparison of sugar-acceptor binding in *Pa*GT3 with that in other plant UGTs

*Pa*GT3 and its isoenzyme *Pa*GT2, which are both UGTs, share the GT-B-fold structure; however, superimposition of the crystal structures of these two UGTs shows many differences (r.m.s.d. of 0.92 for 139 C^α^ atoms; Supplementary Fig. S7*a* ). The loops Phe70–Gly91 (Supplementary Fig. S7*a* , blue box) and Tyr161–Ala200 (Supplementary Fig. S7*a* , black box) around the acceptor-binding pocket in *Pa*GT3 shift outwards compared with the corresponding loops in *Pa*GT2. Similarly, a loop in the C-terminal domain, Pro411–Lys429 (Supplementary Fig. S7*a* , red box), is longer in *Pa*GT3 than in *Pa*GT2. This loop extends up to the opening of the acceptor-binding pocket of *Pa*GT3, while the corresponding loop in *Pa*GT2 is much shorter. As a consequence, the acceptor-binding pocket in *Pa*GT3 is much wider than that in *Pa*GT2, which allows the binding of acceptors in different orientations in *Pa*GT3 compared with *Pa*GT2 to form multiple glycosylated products. This assumption correlates with the previous observation of lower product regioselectivity in *Pa*GT3 compared with *Pa*GT2 using the same compounds (Noguchi *et al.*, 2009 ▸). Also, the wider acceptor-binding pocket in *Pa*GT3 enables the enzyme to glycosylate larger molecules such as capsaicin and betanidin which are not glycosylated by *Pa*GT2. On the other hand, the smaller acceptor-binding pocket in *Pa*GT2 could be a reason why the enzyme is able to glycosylate smaller molecules such as *p*-hydroxybenzoic acid and hydroquinone that are not glycosylated by *Pa*GT3.

Although the acceptor-binding pockets in plant UGTs are usually hydrophobic, sugar acceptors are also stabilized by hydrogen bonds between the acceptors and enzyme residues (Supplementary Fig. S8). In *Vv*GT1 from *V. vinifera*, Ser18 forms a hydrogen bond to the O4 atom of quercetin (Offen *et al.*, 2006 ▸). Similarly, Gln84, His150 and Gln188 form hydrogen bonds to the hydroxyl groups on C7, C3′ and C4′, respectively. In UGT78K6 from *C. ternatea*, Asp367, Asp181 and the main-chain carbonyl O atom of Pro78 stabilize kaempferol, forming hydrogen bonds to the C5, C7 and C4′ hydroxyl groups of the acceptor molecule, respectively (Hiromoto *et al.*, 2015 ▸). Also, in *Pa*GT2 it has been shown that the 3′-OH and 4′-OH groups of the piceatannol molecule are stabilized by hydrogen bonds to His81 and Glu82, respectively (Maharjan, Fukuda, Shimomura *et al.*, 2020 ▸). However, in *Pa*GT3 the acceptor capsaicin is mainly stabilized by hydrophobic interactions and the only possible hydrogen bond is observed to the catalytic His20. On the other hand, the binding of kaempferol is assisted by a network of hydrogen bonds provided by water molecules, which could be due to the smaller size of kaempferol compared with the size of the acceptor-binding pocket.

Comparison of the *Pa*GT3 structure with some other UGTs (Supplementary Fig. S7) shows that the Pro411–Lys429 loop is much longer in *Pa*GT3 than in other UGTs. The Pro411–Lys429 loop in *Pa*GT3 extends to the opening of the acceptor-binding pocket. In other UGTs the corresponding loops are comparatively short and do not extend to the acceptor-binding pocket. Therefore, we assume that the Pro411–Lys429 loop in *Pa*GT3 could play a role in modulating acceptor recognition in *Pa*GT3.

Polyphenols are plant secondary metabolites that have important roles in plant growth and in ensuring their survival in the environment (Kuhn *et al.*, 2016 ▸). Capsaicin, a pungent compound produced by plants of the genus *Capsicum*, behaves as an allelochemical. Capsaicin is likely to be involved in inhibition of the germination of other competiting plants (Kato-Noguchi & Tanaka, 2003 ▸) and is lethal to certain insects (Ahn *et al.*, 2011 ▸). Although humans have long been using capsaicin as a component of spices, recent studies have shown that capsaicin has cardioprotective, antibacterial, anti-inflammatory, anticancer and antioxidant functions. Similarly, kaempferol, along with other flavonoids, is known to protect plants with its antioxidant properties (Shimoji & Yamasaki, 2005 ▸) as well as to induce pollen-specific gene products (Pourcel & Grotewold, 2009 ▸). Kaempferol and its glucosides are also known to have various health benefits such as the prevention of cancer and cardiovascular diseases and to have neuroprotective, antidiabetic, antimicrobial and anti-inflammatory activities (Calderón-Montaño *et al.*, 2011 ▸). Due to the poor water solubility of polyphenols, including capsaicin and kaempferol, it is difficult to administer the amounts of these compounds that are required to have a visible effect. Glycosylation is one of the methods that are used to overcome such problems and the utilization of plant UGTs to glycosylate polyphenols is an economic and environmentally friendly process.

The crystal structure of *Pa*GT3 with capsaicin and UDP-2FGlc provides insight into the capsaicin recognition and glycosylation mechanism of *Pa*GT3. *Pa*GT3 can also glycosylate other long-chain phenolic compounds such as retinol and vitamin E derivatives (Shimoda *et al.*, 2006 ▸), the structures of which are comparable with that of capsaicin. Thus, we assume that *Pa*GT3 utilizes a similar mechanism for the recognition and glycosylation of these compounds as for capsaicin. The crystal structure of *Pa*GT3 with kaempferol and UDP-2FGlc shows that smaller molecules can bind in different positions/conformations due to the large acceptor-binding pocket of the enzyme. The low regioselectivity of glycosylated products with more than one possible glycosylation site could be due to the binding of such molecules in different product-forming conformations. Overall, our crystal structures could be useful to understand the acceptor-recognition mechanism in promiscuous plant UGTs.

## Supplementary Material

PDB reference: *Phytolacca americana* UGT3, complex with kaempferol and UDP-2-fluoroglucose, 7vej

PDB reference: complex with capsaicin and UDP-2-fluoroglucose, 7vek

PDB reference: complex with UDP-2-fluoroglucose, 7vel

Supplementary Tables and Figures. DOI: 10.1107/S2059798322000869/jc5045sup1.pdf

## Figures and Tables

**Table 1:** Macromolecule-production information

Source organism	*Phytolacca americana*
DNA source	pQE30 *Pa*GT3 (Ozaki *et al.*, 2012 ▸)
Forward primer†	5′-CTTTATTTCCAGGGTATGGGTGCTGAACCTCAACAG-3′
Reverse primer†	5′-AGCAGAGATTACCTAAGCATGATAACCCCTCAACTCCTC-3′
Cloning vector	pQE30
Expression vector	pCold I
Expression host	*Escherichia coli* BL21 (DE3)
Complete amino-acid sequence of the construct produced‡	MNHKVHHHHHHLQENLYFQGMGAEPQQLHVVFFPIMAHGHMIPTLDIARLFAARNVRATIITTPLNAHTFTKAIEMGKKNGSPTIHLELFKFPAQDVGLPEGCENLEQALGSSLIEKFFKGVGLLREQLEAYLEKTRPNCLVADMFFPWATDSAAKFNIPRLVFHGTSFFSLCALEVVRLYEPHKNVSSDEELFSLPLFPHDIKMMRLQLPEDVWKHEKAEGKTRLKLIKESELKSYGVIVNSFYELEPNYAEFFRKELGRRAWNIGPVSLCNRSTEDKAQRGKQTSIDEHECLKWLNSKKKNSVIYICFGSTAHQIAPQLYEIAMALEASGQEFIWVVRNNNNNDDDDDDSWLPRGFEQRVEGKGLIIRGWAPQVLILEHEAIGAFVTHCGWNSTLEGITAGVPMVTWPIFAEQFYNEKLVNQILKIGVPVGANKWSRETSIEDVIKKDAIEKALREIMVGDEAEERRSRAKKLKEMAWKAVEEGGSSYSDLSALIEELRGYHA

† The start and termination codons are underlined in the forward and reverse primers, respectively.

‡ The Tobacco etch virus (TEV) protease recognition site is underlined.

**Table 2:** Crystallization

Method	Hanging-drop vapour diffusion
Plate type	Sample cups and siliconized cover glasses
Temperature (K)	293
Protein concentration (mg ml^−1^)	10
Buffer composition of protein solution	20 m*M* Tris–HCl pH 7.5, 100 m*M* NaCl, 5 m*M* DTT
Composition of reservoir solution	0.15–0.20 *M* potassium acetate, 20%(*w*/*v*) PEG 3350
Volume and ratio of drop	1 µl:1 µl
Volume of reservoir (µl)	400

**Table 3:** Data collection and processing Values in parentheses are for the outer shell.

	*Pa*GT3 + UDP-2FGlc	*Pa*GT3 + kaempferol + UDP-2FGlc	*Pa*GT3 + capsaicin + UDP-2FGlc
X-ray source	BL44XU, SPring-8	BL44XU, SPring-8	BL44XU, SPring-8
Detector	EIGER X 16M	EIGER X 16M	EIGER X 16M
Wavelength (Å)	0.9	0.9	0.9
Space group	*P*2_1_2_1_2_1_	*P*2_1_2_1_2_1_	*P*2_1_2_1_2_1_
*a*, *b*, *c* (Å)	94.2, 103.8, 110.0	93.8, 103.1, 108.8	94.2, 103.0, 109.6
Resolution range (Å)	50.00–2.20 (2.26–2.20)	50.00–1.85 (1.91–1.85)	50.00–2.60 (2.71–2.60)
Total No. of reflections	369985	600931	225962
No. of unique reflections	55339 (5439)	89096 (8762)	33386 (3286)
*R*_merge_ (%)	5.1 (65.8)	5.3 (44.6)	7.3 (59.9)
*R*_meas_ (%)	5.5 (71.3)	5.8 (48.2)	7.9 (64.7)
〈*I*/σ(*I*)〉	21.10 (2.96)	19.70 (3.46)	17.86 (3.12)
CC_1/2_	(0.85)	(0.92)	(0.89)
Completeness (%)	99.9 (100)	98.2 (97.8)	99.5 (99.3)
Multiplicity	6.7 (6.8)	6.7 (7.0)	6.8 (7.1)

**Table 4:** Structure solution and refinement Values in parentheses are for the outer shell.

	*Pa*GT3 + UDP-2FGlc	*Pa*GT3 + kaempferol + UDP-2FGlc	*Pa*GT3 + capsaicin + UDP-2FGlc
Resolution range (Å)	48.65–2.20 (2.25–2.20)	47.14–1.85 (1.89–1.85)	45.2–2.60 (2.69–2.60)
*R*_work_/*R*_free_ (%)	20.1/22.4 (31.2/37.1)	20.3/23.6 (26.5/29.6)	20.7/25.9 (31.1/38.2)
R.m.s.d., bond lengths (Å)	0.008	0.015	0.009
R.m.s.d., angles (°)	1.543	1.840	1.030
Ramachandran plot (%)			
Favoured	96.59	97.36	97.63
Allowed	3.41	2.64	2.37
Outliers	90.00	0.00	0.00
Average *B* factors (Å^2^)			
Protein	57.4	41.0	56.6
Ligand	44.8	36.8	52.6
Ion	44.4	29.1	46.5
Water	47.6	41.9	—
PDB code	7vel	7vej	7vek
