# Supplementary material for: Structural basis for substrate recognition in the Phytolacca americana glycosyltransferase PaGT3
Source: Acta Crystallogr D Struct Biol. 2022 Feb 21;78(Pt 3):379–89. doi: 10.1107/S2059798322000869 (PMC8900826; doi:10.1107/S2059798322000869)
Supplement: Supplementary file 1 [file d-78-00379-sup1.pdf]

STRUCTURAL
BIOLOGY

**Volume 78 (2022)**

**Supporting information for article:**

# Structural basis for substrate recognition in *Phytolacca americana* glycosyltransferase *Pa*GT3

**Rakesh Maharjan, Yohta Fukuda, Taisuke Nakayama, Toru Nakayama, Hiroki Hamada, Shin-ichi Ozaki and Tsuyoshi Inoue**

**Table S1** Distances from potassium ion to the carbon and oxygen atoms of 18-crown-6 ether present in *Pa*GT3/UDP-2FGlc/capsaicin crystal structure.

| **Atoms** | **Distance (Å)** | **Average (Å)** | **Atoms** | **Distance (Å)** | **Average (Å)** |
|---|---|---|---|---|---|
| $K^+$-C1 | 3.8 | 3.6 | $K^+$-O1 | 3.3 | 2.9 |
| $K^+$-C2 | 3.7 | | $K^+$-O2 | 2.7 | |
| $K^+$-C3 | 3.6 | | $K^+$-O3 | 2.9 | |
| K-C4 | 3.3 | | $K^+$-O4 | 3.0 | |
| $K^+$-C5 | 3.8 | | $K^+$-O5 | 2.8 | |
| $K^+$-C6 | 3.5 | | $K^+$-O6 | 2.7 | |
| $K^+$-C7 | 3.6 | | | | |
| $K^+$-C8 | 3.7 | | | | |
| $K^+$-C9 | 3.6 | | | | |
| $K^+$-C10 | 3.6 | | | | |
| $K^+$-C11 | 3.5 | | | | |
| $K^+$-C12 | 3.4 | | | | |

**Table S2** The nearest distances between *Pa*GT3 residues in acceptor-binding pocket and capsaicin.

| ***Pa*GT3 residue (atom)** | **Atoms on capsaicin** | **Distance (Å)** |
|---|---|---|
| His20 (NE2) | O10 | 3.3 |
| Met125 (SD) | C13 | 3.5 |
| Phe126 (CZ) | C4 | 4.4 |
| His145 (CB) | C13 | 4.3 |
| Thr147 (CB) | C13 | 3.9 |
| Leu155 (CD1) | C17 | 4.2 |
| Val158 (CB) | C44 | 4.0 |
| Arg159 (CB) | C44 | 3.8 |
| His164 (CE1) | C40 | 4.6 |
| Leu190 (CD2) | C36 | 3.5 |
| Pro191 (CG) | C30 | 4.3 |
| Val194 (CG2) | C37 | 3.7 |
| Leu206 (CD1) | O23 | 4.5 |
| Ala393 (CB) | C6 | 4.0 |
| Glu394 (OE2) | O12 | 3.2 |
| Tyr397 (OH) | C33 | 3.8 |
| Trp417 (NE1) | C24 | 3.7 |

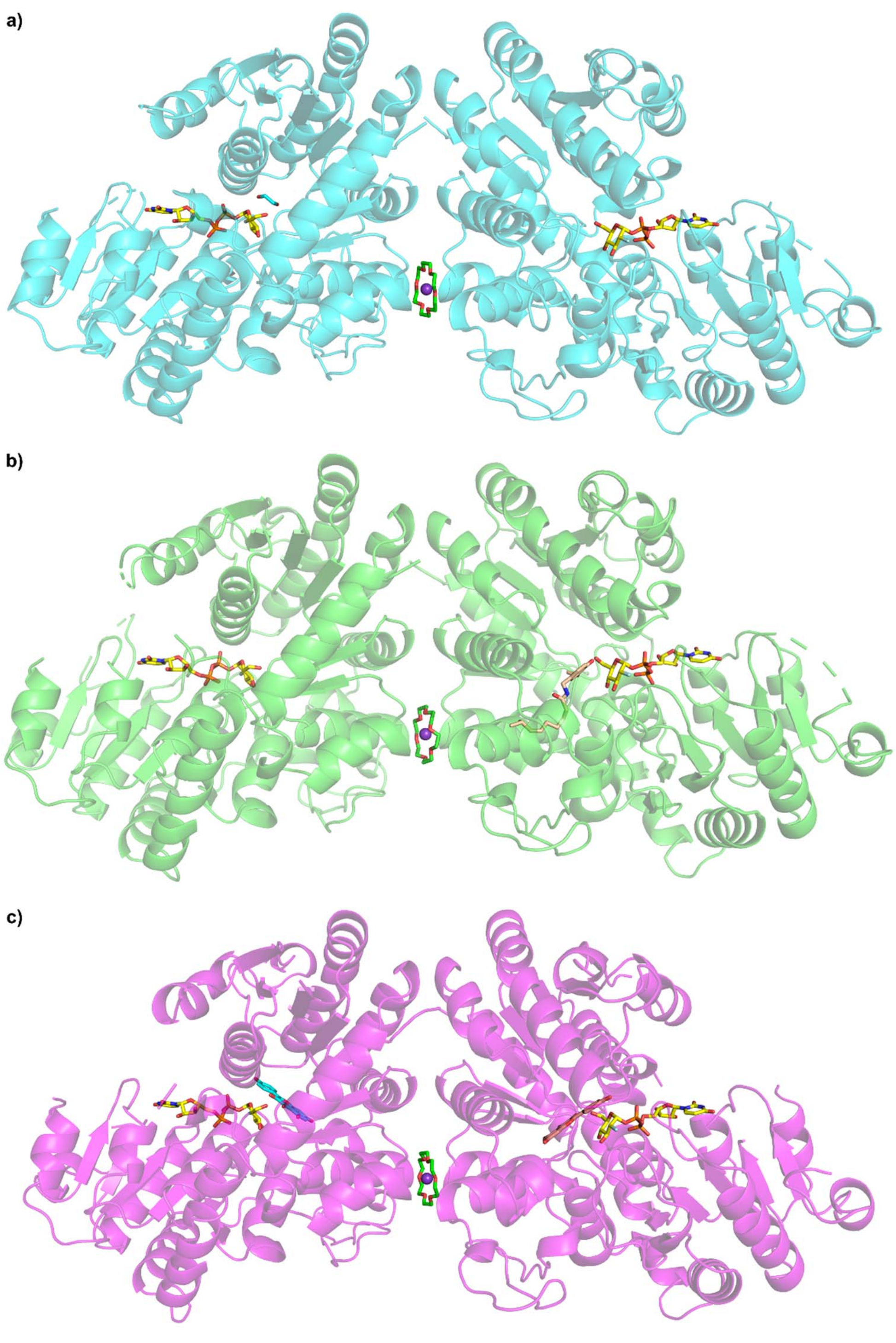


**Figure S1** *Pa*GT3 Structures in asymmetric units. Overall crystal structures of (a) *Pa*GT3 (cyan) with UDP-2FGlc, (b) *Pa*GT3 (green) with UDP-2FGlc and capsaicin, and (c) *Pa*GT3 (magenta) with UDP-2FGlc and kaempferol. There are two molecules of the enzyme in the asymmetric unit. UDP-2FGlc (yellow) is present in all *Pa*GT3 molecules in the crystal structures. Capsaicin (wheat) is present in only one and kaempferol (cyan and salmon) in both molecules of the enzyme in the respective crystal structures. A 18-crown-6 ether (green) with a $K^+$ in its cavity is present between the two molecules of the enzyme.

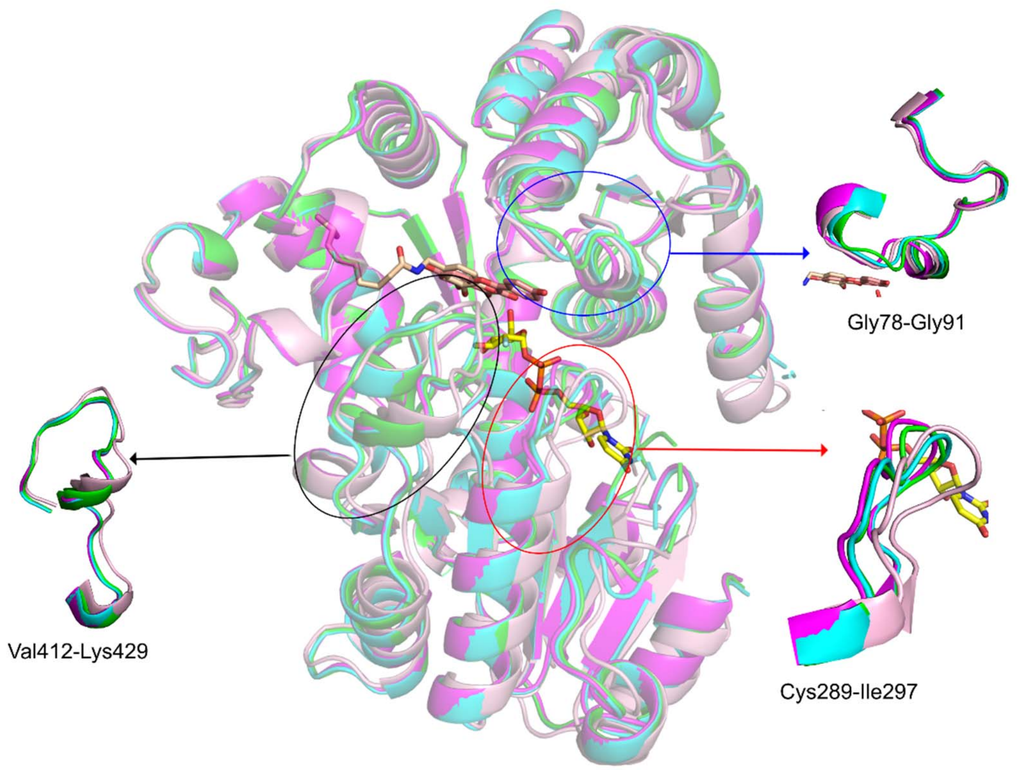


**Figure S2** Comparison of apo- and substrate bound *Pa*GT3 crystal structures. Structural superimposition of ligand bound *Pa*GT3 structures shows high similarity, however loops at the opening of the active-site show subtle differences in presence of different substrates. (apo-*Pa*GT3: pink, *Pa*GT3+UDP-2FGlc: Cyan, *Pa*GT3+UDP-2FGlc+capsaicin: green, *Pa*GT3+UDP-2FGcl+kaempferol: magenta, UDP-2FGlc: yellow, capsaicin: wheat, and kaempferol: salmon).

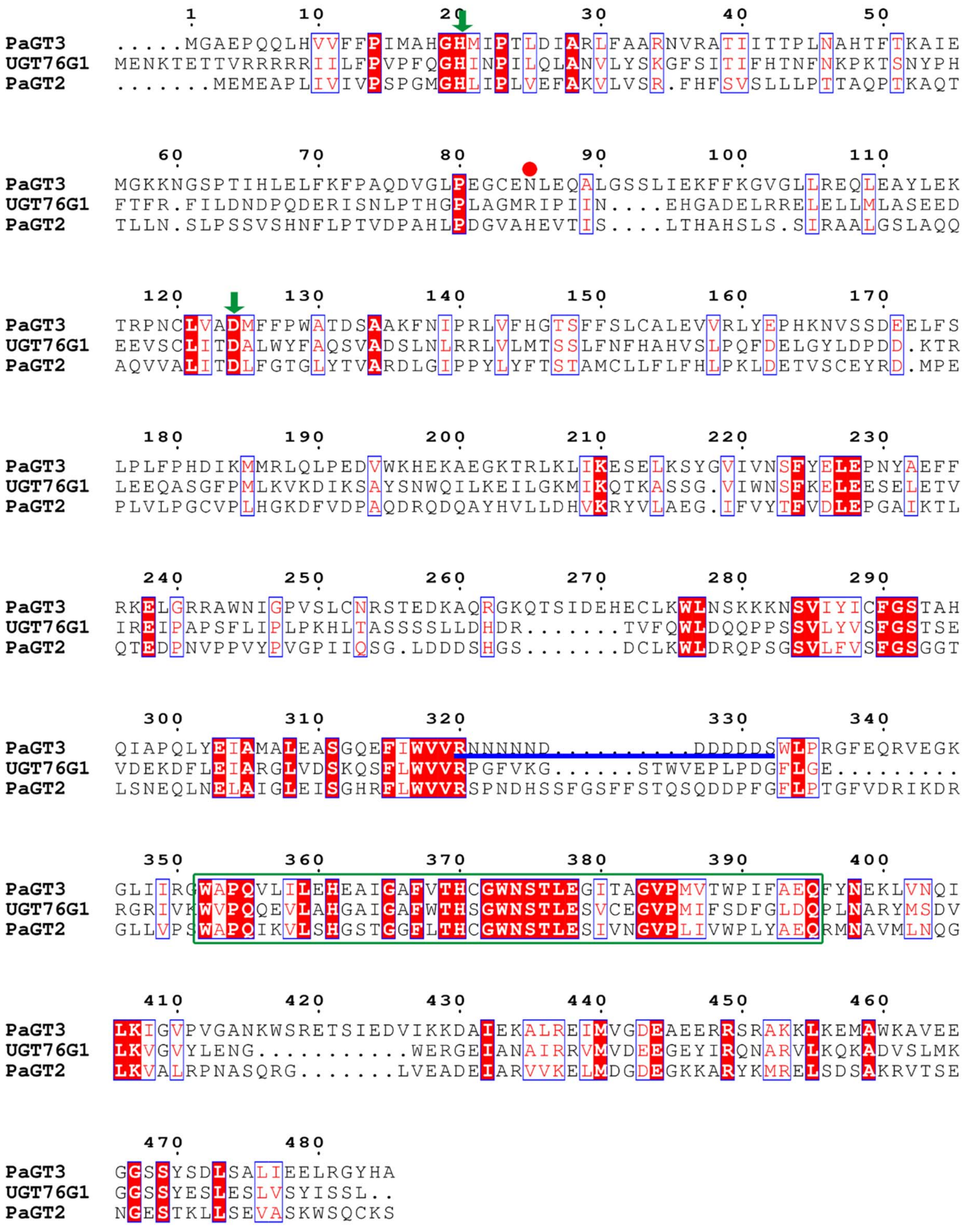


**Figure S3** Amino acid sequence alignment of *Pa*GT3 with UGT76G1 and *Pa*GT2. The conserved catalytic His-Asp pair is indicated with green arrow and the PSPG motif is enclosed in a green rectangular box. The highly polar region in *Pa*GT3 which is disordered in the crystal structures, is underlined (blue). The alternative catalytic histidine (His81) in *Pa*GT2 is indicated with a red circle.

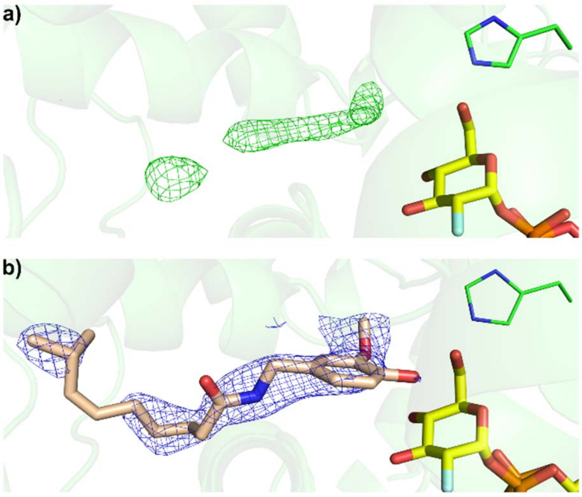


**Figure S4** Electron density maps observed for capsaicin in *Pa*GT3 molecule B. (a) m$F_o$-D$F_c$ omit map (green mesh) observed for capsaicin at 3 σ. (b) Sigma weighted 2m$F_o$-D$F_c$ electron density map (blue mesh) observed for capsaicin.

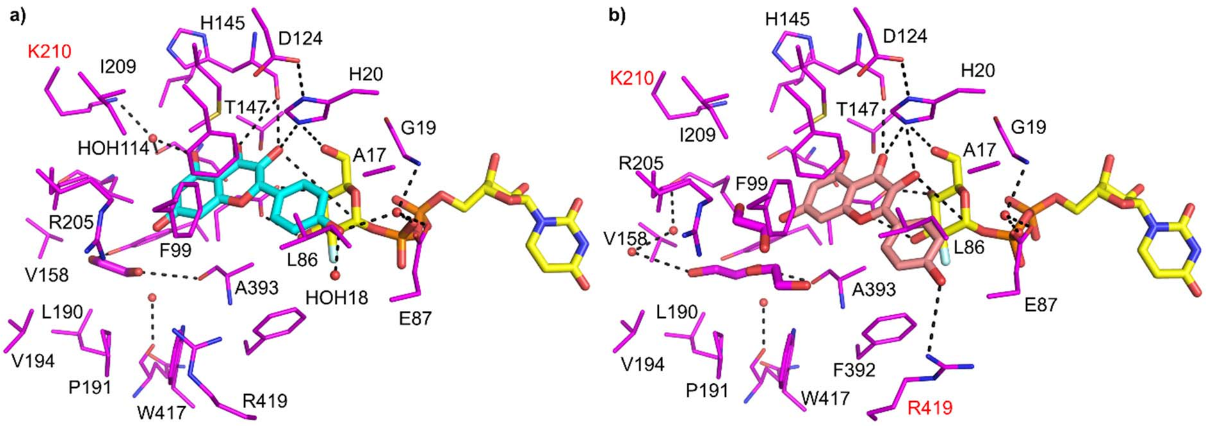


**Figure S5** Interactions of kaempferol in (a) molecule A and (b) molecule B with the residues of *Pa*GT3 in the crystal structure clearly shows that the kaempferol binds in two different orientations in two molecules of the enzyme. As a consequence of different orientations of kaempferol molecules, the sidechain of Arg419 (labelled red) which does not interact with kaempferol in molecule A shifts towards kaempferol and displaces a water molecule to form a hydrogen bond with kaempferol in molecule B. Lys210 (labelled red) forms a water mediated hydrogen bond with the 5OH of kaempferol whereas no such hydrogen bond is seen in molecule B. Also, kaempferol in molecule A forms a hydrogen bond with a water molecule (HOH3), whereas kaempferol in molecule B is seen far from the corresponding water molecule (HOH5) in molecule B.

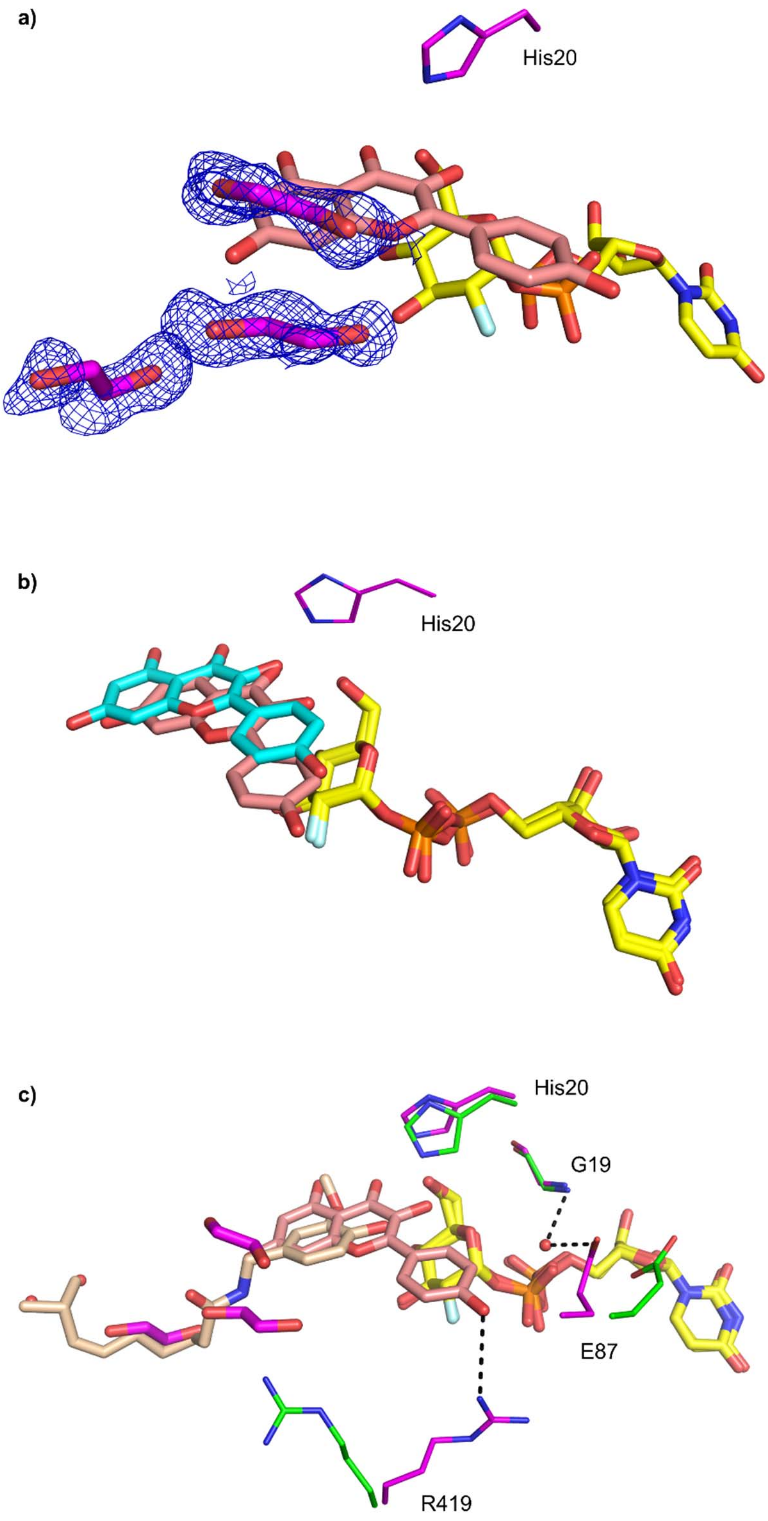


**Figure S6** (a) The acceptor binding pocket in kaempferol complexed *Pa*GT3 is also occupied by ethylene glycol molecules from cryoprotectant solution. The 2m$F_o$-D$F_c$ electron density map observed at 1 σ is shown in blue mesh. The *Pa*GT3 molecule is rotated compared to other figures for clearer view of the electron density map. (b) Comparison of kaempferol in molecule A and B in the *Pa*GT3 crystal structure asymmetric unit shows that the kaempferol in molecule B (salmon) shifts

towards the opening of the active-site than kaempferol in molecule A (cyan). (c) Superimposition of capsaicin and kaempferol in the molecule B of the respective crystal structures show that the glycosylation site on capsaicin lies in the similar position as the glycosylation site on kaempferol. The large acceptor binding site in the kaempferol bound structure is filled with ethylene glycol and water molecules which overlaps with different parts of long alkyl chain of capsaicin. Due to movement of loops towards the active-site in the smaller kaempferol bound structure, Glu87 shifts closer to the substrate. In addition, the sidechain of Arg419 flips to form a hydrogen bond with kaempferol molecule.

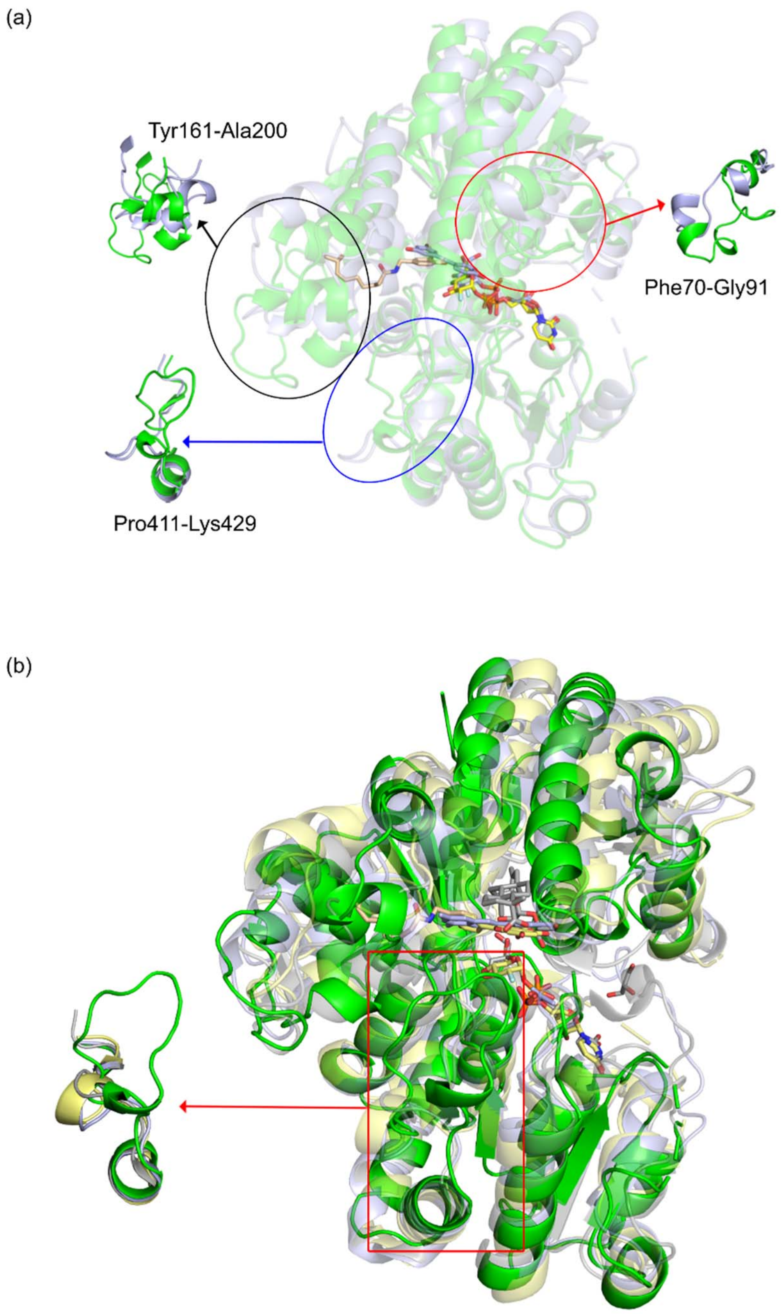


**Figure S7** (a) Comparison of *Pa*GT3 and *Pa*GT2. Both *Pa*GT3 and *Pa*GT2 (light blue) has conserved GT-B fold structure. However, loops around acceptor pocket in *Pa*GT3 are longer compared to *Pa*GT2. Loop region numbers are indicated for *Pa*GT3. (b) Structural comparison of *Pa*GT3 structure with some UGTs. Superimposition of *Pa*GT3 with UGT76G1 (light magenta), *Vv*GT1 (light yellow), and UGT78K6 (light cyan) shows similar structures. However, the C-terminal loop (Pro411-Lys429) in *Pa*GT3 is comparatively longer in *Pa*GT3.

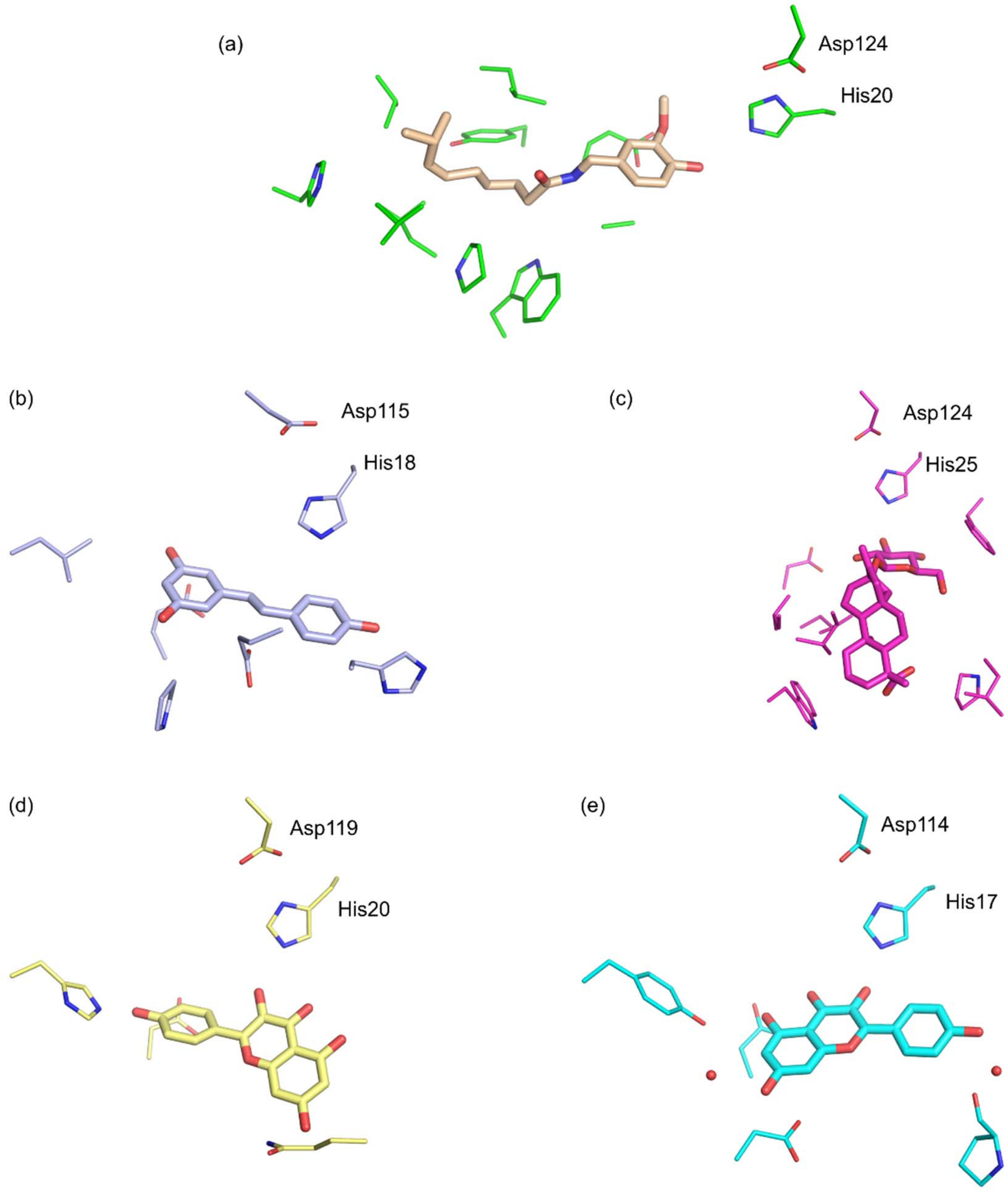


**Figure S8** Comparison of acceptor-binding sites in some plant UGTs. (a) *Pa*GT3, (b) *Pa*GT2, (c) UGT76G1, (d) *Vv*GT1, and (e) UGT78K6 showing the highly conserved His-Asp catalytic pair. The conserved catalytic pair is labelled. It is seen that the formation of elongated cavity allows *Pa*GT3 to bind larger sugar-acceptors such as capsaicin.
